# Comparison of the Choice Effect and the Distance Effect in a Number-Comparison Task by fMRI

**DOI:** 10.1371/journal.pone.0021716

**Published:** 2011-06-30

**Authors:** Yousuke Ogata, Takahiro Horaguchi, Noriya Watanabe, Miyuki Yamamoto

**Affiliations:** 1 Comprehensive Human Science, University of Tsukuba, Tsukuba, Ibaraki, Japan; 2 Physiology, Center for Medical Science, Ibaraki Prefectural University of Health Science, Inashiki, Ibaraki, Japan; 3 Graduate School of Engineering, Tamagawa University, Tokyo, Japan; French National Centre for Scientific Research, France

## Abstract

Behavioral and neurophysiological studies of numerical comparisons have shown a “distance effect,” whereby smaller numerical distances between two digits are associated with longer response times and higher activity in the parietal region. In this experiment, we introduced a two-choice condition (between either the smaller/lower or the larger/higher of two digits) and examined its effect on brain activity by fMRI. We observed longer response times and greater activity with the choice of smaller numbers (“choice effect”) in several brain regions including the right temporo–parietal region, (pre)cuneus, superior temporal sulcus, precentral gyrus, superior frontal gyrus, bilateral insula, and anterior cingulate cortex. These regions correspond to areas that have been suggested to play a role in attentional shift and response conflict. However, brain activity associated with the distance effect disappeared even though the behavioral distance effect remained. Despite the absence of the distance effect on brain activity, several areas changed activity in relation to response time, including regions that were reported to change activity in both a distance effect and a reaction-time-related manner. The result suggested that the level of task load may change the activity of regions that are responsible for magnitude detection.

## Introduction

When human subjects compare the values of two numbers in number- comparison tasks, the relationship between response time (RT) and numerical distance (the difference between the two numbers) is inverse, irrespective of the number of words, Arabic numerals, or number of objects constituting each number. In other words, recognition of a small distance (SD) between two numbers (and quantities) requires more time than does recognition of a large distance (LD). This phenomenon is referred to as the distance effect [1], [2].

Consistent with psychological findings, several neuroimaging studies have revealed that activity changes in the parietal cortex are significantly modulated by the magnitude of the numerical distance [3], [4], [5], [6], [7], [8], [9], [10], with greater activation during the processing of SD than of LD. Clinical studies of patients with lesions of the parietal lobes have also demonstrated the importance of the parietal cortex in numerical manipulation [11], [12], [13], [14], [15], [16]. Results from studies using tasks involving choosing the larger number have shown that repetitive transcranial magnetic stimuli (rTMS) delivered to the left parietal scalp site induced longer RTs only in SD condition but not in LD condition suggesting that the parietal cortex is involved in comparisons of magnitudes [17].

In the same number comparison task, the effect of choice has been described in relation to the magnitude of numbers. Well-documented effect was called spatial numerical association of response codes (SNARC) effect [18]. In a binary response setting, it has been found that relatively small numbers are reacted to faster with the left hand than with the right hand. This SNARC effect is thought to originate from the fact that the mental number line is oriented from left to right (in the case of left-right reading cultures), so that there is congruity between small numbers and left-side responses and between large numbers and right-side responses.

However, unlike the distance effect, the effect of choosing between a larger and smaller object has not been extensively studied from a neurophysiological perspective. Dehaene [2] showed that RTs were significantly longer when choosing smaller than when choosing larger numbers. His result was supported by Horaguchi et al. [19] who used near-infra red spectrometry (NIRS) as a neuroimaging technique for identifying the brain regions responsible for the choice effects associated with a number-comparison task involving Arabic numerals. They detected a difference between the two choice conditions (the smaller digit choice: SC vs. the larger digit choice: LC) and showed that the activity in the right temporo-parietal region was higher under the SC condition. However, they could not detect a difference between the two distance conditions (SD vs. LD). Due to the limitation of NIRS measurements, they could not identify neuronal processes that were occurring during the task.

A number of studies, including those using unit recording in monkey brains [20], [21], [22] and fMRI in humans [3], [4], [8], [10], [23], [24], [25], clearly show that the inferior parietal region is involved in numerical processing. The involvement of this region is also shown in other quantitative information processing such as physical sizes or luminance comparison [6], [10], [26], [27], [28]. In monkey parietal cortex, Sawamura et al. [22] reported that the number selective cells and those that responded to task-related cues that had no numerical component were found within the same area.

In addition to the processing of numerical quantity of multiple modalities, the interaction of multiple functions of IPS have also been suggested such as, reaction time [29], time and space perception [30], [31], and attention [32], [33], [34], [35].

Gobel et al. [29] argued that the activation of the IPS during magnitude comparison may be related to response-selection rather than number-specific processing, and these 2 functions might be interacting in the IPS [29].

Based on our previous NIRS results [19] we hypothesized that it might be possible to observe the interaction between numerical processing and other functions if we use the same modality (Arabic numbers) but change the decision process. By adding two-choice conditions (to choose either the larger one or the smaller one, instead of choosing merely the larger one) would change neuronal activity associated with the distance effect. It might give us a clue whether the higher levels of activity observed in the parietal region during the number comparison task are solely attributable to numerical processing or represent more general activities, such as attention [36] or reaction time [29].

## Methods

### Subjects

Thirteen healthy volunteers participated in the fMRI study (nine males, four females; average age: 21.7; all right handed). The study protocol was approved by the institutional (AIST and University of Tsukuba) ethics committees and conformed to the ethical standards contained in the 1964 Declaration of Helsinki. All subjects provided informed consent prior to their participation in the study.

### Stimuli

Pairs of numerical numbers (black) with visual angles of 1.47°×0.73° (height×width) were presented as stimuli; the margins from the center to the inside and outside of each digit were 1.27° and 2.00°, respectively. The stimuli were presented on a screen in the fMRI experiment. A red fixation point (diameter approximately 0.57° visual angle) was displayed at the center of the monitor throughout the experiment to eliminate eye movement and related brain activity. The instructions for the choice required in each task were presented in Japanese on the screen before the beginning of each session. The pairs of digits were divided into two categories of numerical distance: small distance (SD) and large distance (LD). The SD pairs included distance sizes (D) 1, 2, and 3 (total of 18 pairs). D = 1 pairs included 1–2, 2–1, 3–4, 4–3, 6–7, 7–6, 8–9, and 9–8; D = 2 pairs included 1–3, 3–1, 4–6, 6–4, 7–9, and 9–7; and D = 3 pairs included 1–4, 4–1, 6–9, and 9–6. LD included distance sizes 5, 6, and 7 (total of 18 pairs). D = 5 pairs included 1–6, 6-1, 2–7, 7–2, 3–8, 8–3, 4–9, and 9–4; D = 6 pairs included 1–7, 7–1, 2–8, 8–2, 3–9, and 9–3; and D = 7 pairs included 1–8, 8–1, 2–9, and 9–2. Extreme values such as 1 or 9 were displayed with equal frequency in both distance pairs, and the middle number (5) was never displayed. Each pair was displayed only once within a block.

### Task Design

**Figure 1:**
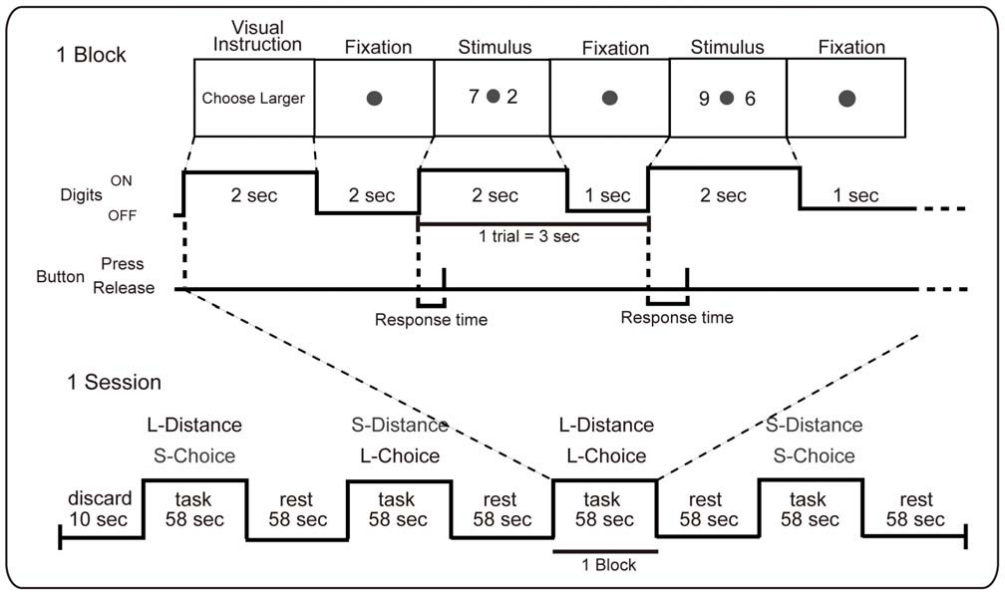
Task sequence of fMRI sessions. The session for each task included an instruction period, a resting period (in which only a fixation point was displayed), and a task period. Subjects were instructed to stare at a fixation point throughout the session and to select the correct digit as quickly as possible after a pair of digits was displayed. Instructions about which digit to select (larger/smaller) were visually presented before the beginning of each block (fMRI task).

Participants were instructed to compare two digits, which were displayed on a screen and viewed through a prism mirror within a MRI scanner, and to choose either the larger or the smaller digit. The task program was controlled by E-prime (Psychology Software Tools, Inc., Pittsburgh, PA, USA). We used a block design, and the task sequence is shown in Figure 1.

One session contained four blocks, each of which consisted of one combination of the two distance conditions (18 LD or 18 SD pairs)×2 choice conditions (LC or SC). Each pair appeared only once per block, and the order of blocks was randomized. Blocks were separated by the presentation of a 58-sec fixation point. At the beginning of each session, the fixation point was presented for 10 sec. Task switching of choice was introduced between blocks, and the order of choice was randomized across subjects. One block was composed of a 2-sec initial presentation of the instructions (“Choose the larger/smaller digit”), a 2-sec fixation point, repeated (18 times) presentations of pairs of digits (2 sec), and the fixation point again (1 sec) (in total, 4 sec+3 sec×18 = 58 sec). Stimulus presentation was set to 2 sec, as response times sometimes exceeded 1 sec but never exceeded 2-sec in preliminary studies. After the presentation of each pair of digits, subjects were asked to respond as quickly as possible by using their second or third finger to press the one of the two buttons on the response pad (MRI-compatible Joystick, Resonance Technology, Inc., Los Angeles, CA, USA) that corresponded to the side on which the correct digit appeared. The stimulus disappeared after 2 sec even when participants did not respond. Each subject received two sessions: in one, the right hand was used, and in the other, the left hand was used to cancel out any effect of which hand was used. The order of hands was counterbalanced across subjects.

### Behavioral Analysis

RT for each subject under four conditions were statistically analyzed by two-way repeated-measures analysis of variance (ANOVA). The factors used in the ANOVA were distance (SD, LD) and choice (SC, LC).

### fMRI Parameters

A time-course series of 242 volumes (per session) was acquired with T2*-weighted, gradient echo, echo planar imaging (EPI) sequences with a 3.0-T MRI system (Signa Horizon; General Electric Medical Systems, Milwaukee, WI) equipped with a standard birdcage head coil. Each volume consisted of 16–18 slices with a slice thickness of 6.0 mm (2.0-mm gap). Parameters for fMRI were set as for Kowatari et al. [37]. The TR was 2000 ms, the TE was 30 ms, and the flip angle was 70°. The digital in-plane resolution was 64×64 pixels. The first five volumes were discarded to stabilize magnetization. For anatomical information, high-resolution T2-weighted images of the same slices of EPI scans were acquired with a spin echo sequence, with a 20-cm field of view (256×256 matrix, 16–18 slices, TR 5,000 ms, TE 70 ms).

### fMRI Analysis

The image data were analyzed using Statistical Parametric Mapping 5 (SPM5; Welcome Department of Imaging Neuroscience, London, UK; http://www.fil.ion.ucl.ac.uk/spm) implemented in MATLAB (MathWorks, Natick, MA). To correct for the head motions of each subject during MRI, the images were realigned to the first EPI volume. All the EPI volumes were then co-registered with high-resolution T2-weighted images of the same slices of EPI scans, and all volumes were spatially normalized to the SPM5 template (Montreal Neurological Institute: MNI) space. Subsequently, all normalized images were smoothed using an isotropic Gaussian kernel (8 mm^3^ full-width at half-maximum) to increase the signal/noise ratio in the images. A 128-sec temporal high-pass filter was applied to the data to remove low-frequency baseline drift in the BOLD signal.

In the first-level analysis, the fMRI signal obtained from each subject during each session was fitted with a hemodynamic response function to detect significant increases from the rest condition. *T*-statistic maps were acquired from each subject for the four conditions: LC/LD, LC/SD, SC/LD, and SC/SD. These four *t*-statistics maps were used in the second-level analysis, for a group comparison using a random-effect model with a two-way ANOVA (distance×choice). The results were reported as p-value with uncorrected for multiple comparison (*P*_unc_), *P*_unc_<0.001 with an extent threshold of >50 voxels.

To compare the effect size of each condition (LC/LD, LC/SD, SC/LD, and SC/SD), averaged BOLD signal intensity of defined areas was calculated using MarsBaR (http://marsbar.sourceforge.net) for 4 conditions.

### Defining Areas that Change Activities in Relation to RT

To elucidate the areas that represent response time on the number-comparison task, we used the average RTs of all subjects under each of four conditions (LC/LD, LC/SD, SC/LD, SC/SD) as the parameter of the contrast vector for second-level analysis. The results were reported as p-value with Family-wise error correction (*P*_FWE_), *P*_FWE_<0.05.

## Results

### Behavioral Results

**Figure 2:**
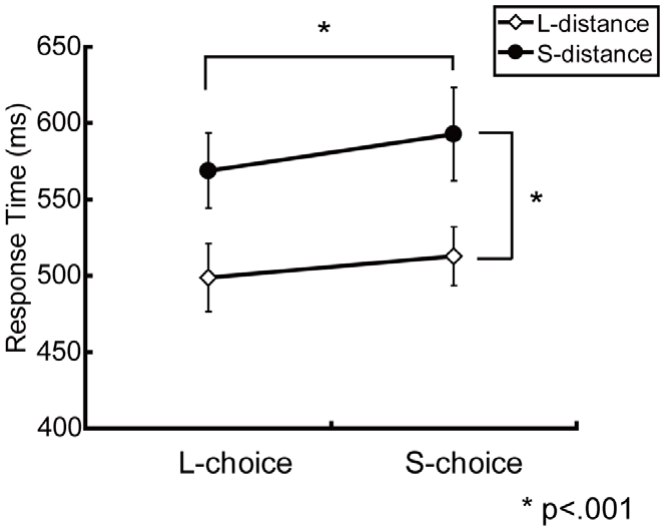
Average response times of 4 conditions. Error bars indicate standard errors of mean. The result of two-way ANOVA showed significant main effects of distance and choice, but no interaction between the two.

Figure 2 shows averaged RT in the fMRI task across subjects. The RT (+S.E.M) under the LC/LD condition was 498.7+22.2 ms; under the LD/SC condition, it was 512.9+19.2 ms; under the LC/SD condition, it was 568.9+24.8 ms; and under the SD/SC condition, it was 592.6+30.6 ms. The results of a two-way ANOVA (Table 1) showed significant main effects for distance (*F*(1,12) = 46.95, *p*<0.001) and choice (*F*(1,12) = 25.70, *p*<0.001), but the interaction between distance and choice was not significant. (*F*(1,12) = 0.40, *p* = 0.54). Error rates for each condition was very low (LC/LD:0.21%; LC/SD:1.07%; SC/LD:0.64%; SC/SD:2.56%) and significant difference was observed in factor of distance (*F*(1,12) = 11.14, *p*<0.01) but no significant difference was observed in choice (*F*(1,12) = 3.6, *p* = 0.08), or the interaction (*F*(1,12) = 1.35, *p* = 0.26) by ANOVA (distance×choice).

**Table 1:** ANOVA table of response time analysis.

Source of variation	Sum of Squares	Degrees of Freedom	Mean Squares	*F*-value	
Subject(S)	347328.0944	12	28944.0079		
Choice(A)	4658.1156	1	4658.1156	25.7	**
SxA	2175.091	12	181.2576		
Distance(B)	72941.5735	1	72941.5735	46.95	**
SxB	18641.9924	12	1553.4994		
AxB	293.0249	1	293.0249	0.39	**
SxAxB	8886.7957	12	740.5663		
Total	454924.6881	51			

**p<0.001.

### Distance Effect and Choice Effect in fMRI

**Figure 3:**
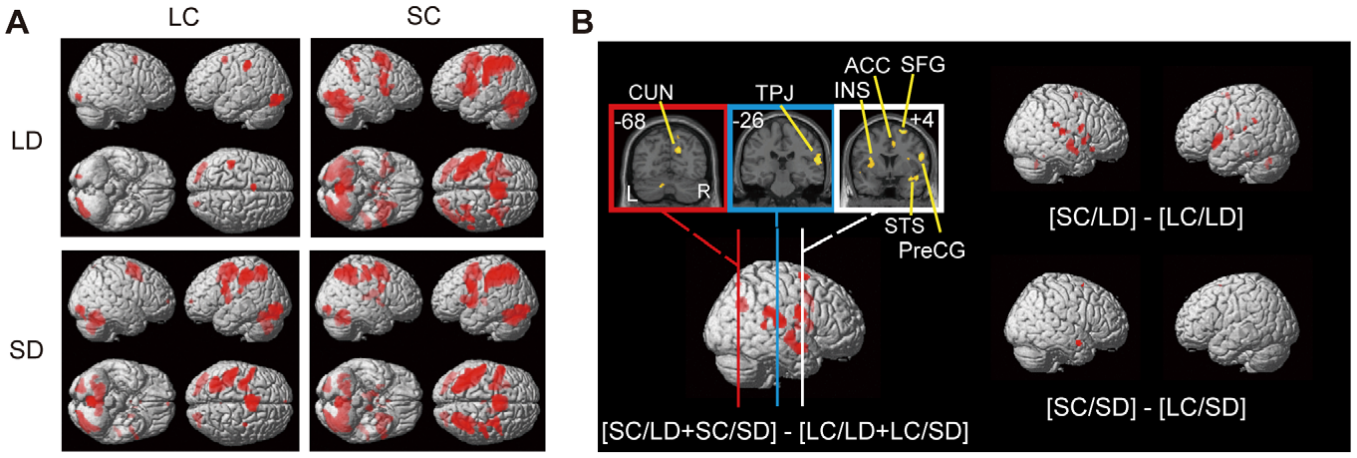
Brain areas activated under each condition. (A) Red areas showed greater activation in the task than in the rest period. LC: the larger digit choice; SC: the smaller digit choice; LD: Large distance; SD: Small distance. The threshold *p*-value under each condition was 0.001 (uncorrected). (B) Comparison between the brain areas that were activated under the SC and LC conditions with *p*-values lower than 0.001 (uncorrected). The number at the upper left of the picture in each section indicates the *y*-level on the MNI coordinates. (CUN: cuneus/precuneus, TPJ: temporo–parietal junction, INS: insula, ACC: anterior cingulate cortex, SFG: superior frontal gyrus, STS: superior temporal sulcus, PreCG: precentral gyrus).

Table 2 summarizes activated regions under each condition; these are also shown in Figure 3a. The brain activities under the four conditions (two choice conditions: LC or SC×two distance conditions: LD or SD) showed significant differences, with less activity occurring under the LC than under the SC condition. In the contrast between the two choice categories (SC(LD+SD) vs. LC(LD+SD)), the left insula, right superior temporal sulcus (STS) extending to the right insula, right temporo–parietal junction (TPJ), right anterior cingulate gyrus (ACC), right (pre)cuneus, right frontal regions (precentral gyrus: PreCG, medial frontal gyrus: MFG and superior frontal gyrus: SFG) demonstrated greater changes in the BOLD signal under the SC condition than under the LC condition (*p*<0.001, uncorrected; Figure 3b and Table 3). On the other hand, no brain region showed a main effect for distance (SD(LC+SC) vs. LD(LC+SC)) or for the choice–distance interaction (*p*<0.001, uncorrected).

**Table 2:** MNI coordinates and statistical details for areas that were activated in each condition.

					coordinates		
Area	side	voxel size	T value	Z value	x	y	z
*LC/LD*							
Inferior occipital gyrus	Left	329	6.26	5.33	−22	−90	−12
Supplemental motor area	Left	100	4.42	4.03	−6	6	54
Postcentral gyrus	Left	205	4.39	4	−44	−32	44
Inferior occipital gyrus	Right	50	4	3.7	24	−92	−8
*LC/SD*							
Lingual gyrus	Left	885	8.13	6.42	−20	−90	−12
Supplemental motor area	Left/Right	1186	6.65	5.57	−6	6	56
Poctcentral gyrus/Superior parietal gyrus	Left	1997	5.64	4.92	−46	−32	50
Vermis	Right	1979	5.12	4.55	4	−66	−18
Lingulai gyrus	Right	269	5.02	4.48	24	−90	−10
Precentral gyrus/Medial frontal gyrus	Left	606	4.42	4.02	−30	−14	56
Insula	Left	435	4.39	4	−46	0	8
Putamen	Left	76	3.79	3.52	−22	−4	12
*SC/LD*							
Lingual gyrus	Left	5643	7.96	6.32	−20	−90	−12
Supplemental motor area	Left/Right	2520	7.44	6.04	−6	6	54
Postcentral gyrus/Superior parietal gyrus	Left	3702	6.19	5.28	−48	−32	50
Insula	Left	2080	5.36	4.72	−40	6	4
Insula	Right	842	4.67	4.22	42	10	2
Superior temporal gyrus	Right	65	4.04	3.73	66	−42	24
Inferior parietal gyrus	Right	435	3.97	3.68	50	−40	58
Precentral gyrus	Right	209	3.92	3.63	36	−8	56
*SC/SD*							
Lingual gyrus	Left	2887	7.29	5.95	−22	−90	−12
Supplemental motor area	Left	1563	6.91	5.73	−6	6	54
Postcentral gyrus/Superior parietal gyrus	Left	3633	6.37	5.4	−48	−32	48
Precentral gyrus/Medial frontal gyrus	Left	2206	5.2	4.61	−46	4	30
Superior parietal gyrus/Inferior parietal gyrus	Right	2570	4.8	4.31	24	−68	50
Lingual gyrus	Right	195	4.55	4.13	22	−90	−10
Caudate/Thalamus	Right	280	4.03	3.72	14	−8	18
Thalamus	Left	103	3.85	3.58	−12	−16	6
Pallidum	Right	54	3.57	3.35	24	−2	6

LC: Choose larger, SC: Choose smaller, LD: Large distance, SD: Small distance. *P_unc_* were all <0.001.

**Table 3:** MNI coordinates and statistical details for areas that activate in contrast [SC – LC].

					coordinates		
Area	side	voxel size	T value	Zvalue	x	y	z
Superior temporal sulcus	right	371	5.06	4.5	46	−2	−18
Superior frontal gyrus	right	75	4.31	3.95	24	6	66
Temporo-parietal junction	right	280	4.29	3.93	66	−22	12
(pre)cuneus	right	161	4.17	3.84	16	−64	34
Insula	left	358	4.05	3.74	−38	12	2
Precentral gyrus/Medial frontal gyrus	right	196	4.02	3.72	54	2	20
Anterior cingulate gyrus	right	109	3.87	3.59	8	8	40

*P_unc_* were all <0.001.

**Figure 4:**
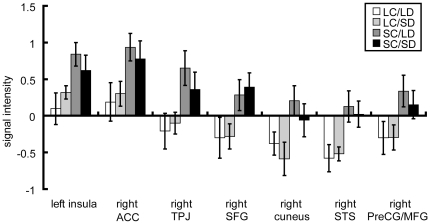
Average BOLD signal change from the rest in areas indicated in **Figure 3b**. Error bar indicates S.E.M. LC: the larger digit choice; SC: the smaller digit choice; LD: large-distance; SD: small-distance.

We quantified the BOLD signal change from the rest among these four conditions in the regions listed above (Figure 4). The result showed two major tendencies. One type of reaction was seen in the Insula and ACC, which showed signal increase in both LC and SC conditions with higher increase in SC. The other type was the decrease of the BOLD signal below the resting state in LC condition, with the increased signal above the rest in SC condition. The latter areas included SFG, TPJ, PreCG/MFG, (pre)cuneus and STG.

### Regions that Changed Activity in Relation to RT

**Figure 5:**
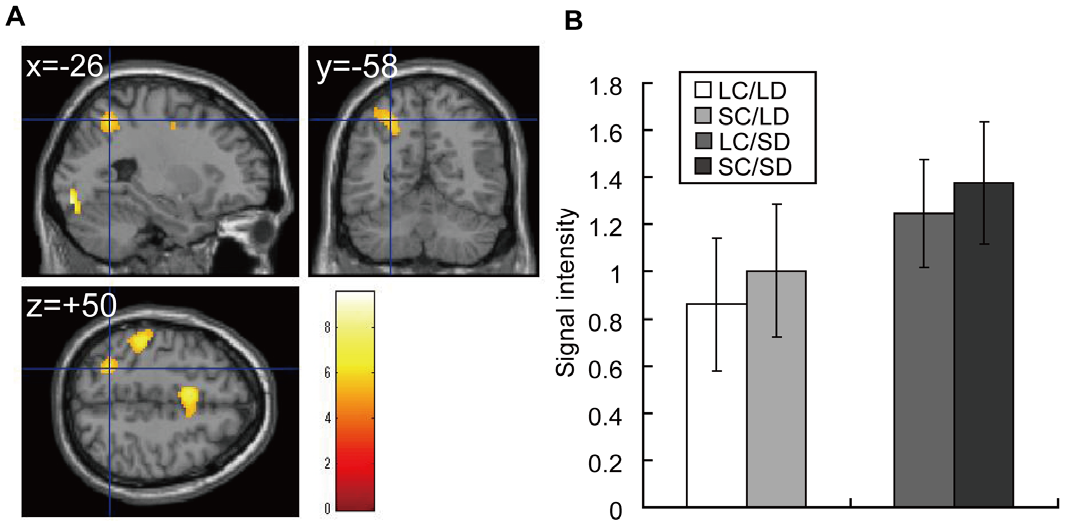
RT-related BOLD signal change. (A) Parametric contrast of regions activated in proportion to the measured RT of each condition (SC/SD>SC/LD>LC/SD>LC/LD) (*p*<0.05, FWE corrected). The hair line indicates intra-parietal sulcus (IPS). (B) BOLD signal intensity at MNI coordinate (−26, −58, 50).

As Pinel et al. [8] and Gobel et al. [29] pointed out, activation associated with numerical-distance judgment cannot be separated from neuronal processes associated with reaction time change. Pinel et al. [8] showed that activation in bilateral IPS and precuneus correlated with the RT in number comparison task. Also in Gobel et al. [29], they compared reaction time and brain activity between the number comparison task and the vertical line detection task and demonstrated that IPS activation varied only with RT changes irrespective of the experimental task. Therefore, we examined areas that changed activity in relation to RT by elucidating areas that changed activity in proportion to the measured RT for each condition (SC/SD>SC/LD>LC/SD>LC/LD: the higher the activity was, the slower the reaction time was). These areas include right IPS, bilateral supplemental motor area, left postcentral sulcus/inferior parietal gyrus, bilateral lingual gyrus, and right cerebellum. These regions did not overlap those that showed higher activity in SC than in LC (Figure 5, Table 4).

**Table 4:** MNI coordinates and statistical details for areas that change activity in relation to RT.

						coordinates		
Area	side	voxel size	T value	Z value	p value (FWE)	x	y	z
Lingual gyrus	Left	445	9.55	7.12	<0.001	−20	−90	−12
Supplemental motor area	Left/Right	660	8.2	6.45	<0.001	−6	6	54
Postcentral sulcus/Inferior parietal sulcus	Left	549	7.21	5.91	<0.001	−46	−32	48
Lingual gyrus	Right	97	6.15	5.25	0.002	24	−90	−10
Intra-parietal sulcus	Left	303	6.03	5.18	0.002	−26	−58	50
Cerebellum	Right	67	5.51	4.83	0.011	4	−68	−20

*P_FWE_* were all <0.05.

## Discussion

### Differential Effects of Distance and Choice in Brain Activity

Our behavioral data confirmed the presence of both the distance effect (longer RTs under the SD compared with the LD condition), and the choice effect (choosing the smaller digit caused a slower RT under both the LD and SD conditions). Behaviorally, RT was longer with SC than with LC, and the fMRI results indicated the choice effect such that the slower the RT was (SC), the higher the BOLD signal change became. However, in contrast to the choice effect, no brain region showed a main effect for distance (SD vs. LD). As no distance effect was observed in brain activity, it is unlikely that brain activity may reflect error rates, because significant difference of error rates was observed only between LD and SD, but not between LC and SC.

These results contradict other imaging studies that have shown activity differences between SD and LD, with higher activity in SD [4], [5], [6], [7], [8], [10], [37]. These other studies showed that the bilateral posterior intra-parietal sulcus (IPS), right precuneus, and right MFG showed higher activity under the SD than under the LD condition. As we used the same combination of 2 digits as other experiments that showed clear BOLD signal change associated with distance effect [4], [5], [6], [7], the absence of distant effect in brain activity was not due to the lower sensitivity of stimuli that we used. Therefore the major difference between ours and other experiments was that their tasks were performed under the instruction “to choose *the larger* number” and did not use the two-choice condition as we did. In our experiment subjects had to switch choices between blocks, the task load was heavier as the task required constant attention to which choice was required.

It seems to be our natural tendency that larger numbers are more salient than smaller ones as shown by Merkley [38], when comparing 2 digits, subjects tend to fixate their gaze more often on larger numbers than on smaller numbers. Therefore, in small choice, subjects need to oppose the natural tendency of choosing larger one, and to do so, more number of neuron are to be recruited and takes longer to decide resulting in longer response time.

In SNARC effect, which is a choice-related phenomenon, the mental number line is oriented from left to right (in the case of left-right reading cultures), so that there is congruity between small numbers and left-side responses and between large numbers and right-side responses [18]. However, in our experiment, we designed the task to cancel the SNARC effect; subjects had to respond by right or left hand first and then performed the same task by switching hand. In addition, the combination of the same 2 digits was shown twice with side reversed (e.g. 3-7 and 7-3). Therefore, the choice effect that we observed may be independent from SNARC effect.

### Relation to Attentional Networks

In the contrast between the two choice categories (SC(LD+SD) vs. LC(LD+SD)), the left insula, right STS extending to the right insula, right ACC, right TPJ, right frontal regions (PreCG,/MFG and SFG) (pre)cuneus, demonstrated greater changes in the BOLD signal under the SC condition than under the LC condition. Among these areas, TPJ, MFG, IPS and (pre) cuneus were described as a part of attentional network in the review by Corbetta et al. [36] and by Behrmann et al. [39]. They described two types of attentional biasing signals; dorsal network or goal-directed (top-down) and ventral network or stimulus-driven (bottom-up). The former is mediated by right MFG/right PreCG, IPS and precuneus [40], [41], [42], [43], and the latter is mediated by the right MFG/PreCG and right TPJ, which is activated independently of the sensory modality and has been implicated in serving a multisensory attentional function [44]. Right hemispheric dominance in attentional function has also been documented [32].

BOLD signal changes in areas that showed higher activation with SC than with LC were shown in Figure 4. Two types of responses were observed; one is associated with the increase of BOLD signal in both SC and LC conditions but with higher activity in SC. The other type showed the decrease of signal intensity from the rest condition in LC but the increase in SC condition. The latter group included right TPJ, right SFG, right PreCG, right STS, and right (pre)cuneus.

It has been suggested that the TPJ coordinates voluntary and stimulus-driven attentional control settings to determine which stimuli effectively compete for attention [45]. In our natural tendency, the larger digit in a pair seems to carry the target-defining feature (i.e., to be more salient); therefore, subjects may have to re-orient their attention each time in the task of choosing the smaller digit. TPJ activation under the SC condition may possibly reflect such an operation.

STS in conjunction with IPL, was also suggested as a part of top-down control system [33] This region was also showed higher activation in SC than in LC condition. Similar explanation might be applicable to (pre)cuneus as several authors reported the involvement of (pre)cuneus in attentional system, for attention shift between two stimulus features [46] and at the appearance of unattended stimulus [47].

Inferior parietal lobule is reported as activated region for distance effect [4], [5], [6], [7], [8], [10], [48] but also as a part of attentional network [33], [34], [35], [42], [49]. It is plausible that in our experiment, because subjects had to pay attention to the choice as well as the distance, the task load was heavier than in a one-choice experiment. If numerical and attentional operations shared the same neuronal resources in the IPS, an increased demand for attention may have used up resources that would otherwise have been available for numerical processing, leaving fewer neurons to participate in the number-comparison task itself. It is possible that the number of neurons that are required for processing numerical information might be sufficient, but in both LD and SD the BOLD signal change becomes weaker than one-choice task and as a floor effect, the difference in signal intensity between SD and LD become undetectable.

A similar phenomenon has been observed by increasing the task load in experiments that used other tasks. Using a duration-discrimination task, Livesey et al. [50] showed that time-related activity in the right IPS, pre-SMA, and parts of the prefrontal cortex disappeared and reversed in polarity as a function of task difficulty, and they suggested that activity in these regions was related to task demand. Based on the observation that a numerical task impaired a time-estimation task under a dual-task paradigm, Walsh also suggested that processing time and quantity (number) share resources in the parietal cortex [31]. Also, Ballan et al. reported that the distance effect was diminished by visual-noise load [51].

It is possible that the difference between LD and SD in IPS may partly represent RT, as Gobel et al. pointed out [29]. In our experiment too, we observed regions that changed activity in parallel to RT, including IPS, lingual gyrus and precentral gyrus (Figure 5). These RT-related areas did not overlap with regions that showed higher activation in SC than in LC. IPS is always activated during response selection [52] and is not restricted to number comparison tasks. Therefore, it is possible that IPS neurons are serving for numerical task, response-time related function and attention.

### Response Conflict and Attention

Insula and ACC both showed increased activity in both SC and LC but with higher activity in SC (Figure 4). A number of fMRI studies have suggested that one function performed by the ACC involves conflict monitoring or error detection [53], [54], [55], [56]. The role of the insula in this context may also be related to conflict. Several researchers confirmed that conflict processing is reliably associated with activation in the anterior insula as well as in the ACC, prefrontal cortex, and parietal cortex [57]. The SFG has been implicated in the resolution of conflict through the top-down posterior attentional system that contains the TPJ [58], [59], [60]. Thus, higher activation of ACC and SFG under the SC condition than the LC condition may reflect the high conflict associated with the selection of a response given that selecting the smaller number is contrary to our natural (conditioned) decision-making. Thus, the ACC and insula may be involved in monitoring the conflict that arises in choosing a smaller number, the SFG may participate in resolving this conflict as well as orienting attention.

### Conclusion

In this study, our results indicated that the choice effect is represented as brain activity. As a behavioral study in monkeys also showed the choice effect [61], it would appear that this effect is innate rather than a product of learning, at least among primates. Animals, including humans, often encounter situations in which they need to choose larger or smaller quantities as quickly as possible. Choosing a larger option, such as an amount of food or the size of a community to follow, would be associated with increased chances of survival. Under the “smaller” choice condition, subjects may have to re-orient their attention from larger to smaller digits (or quantities). Because this represents an unnatural condition for humans or other animals, the situation could cause conflict and require attentional shift, which activated attentional network and conflict-related regions.
